# Bio-based Substances From Compost as Reactant and Active Phase for Selective Capture of Cationic Pollutants From Waste Water

**DOI:** 10.3389/fchem.2020.00550

**Published:** 2020-07-21

**Authors:** Giuliana Magnacca, Flavio Neves Dos Santos, Razieh Sadraei

**Affiliations:** ^1^Dipartimento di Chimica, Università di Torino, Turin, Italy; ^2^Centre for Nanostructured Interfaces and Surfaces (NIS) Interdepartmental Centre, Università di Torino, Turin, Italy; ^3^Faculty of Science and Engineering, University of Wolverhampton, Wolverhampton, United Kingdom

**Keywords:** bio-based substances, alumina, porous monolith, organic/inorganic hybrid, removal of pollutant, cationic molecules

## Abstract

Alumina porous monoliths were successfully fabricated using a simple and reproducible synthesis dispersing gamma alumina phase from commercial boehmite (GAB) in water containing water-soluble bio-based substances (BBSs) obtained from composted biowaste. The wet mixture obtained was shaped in form of small spheres and then dried and calcined at 500°C in order to burn the organic matter and obtain mesoporous monoliths. They were successively functionalized with BBSs in order to introduce BBS functional groups and obtain an efficient adsorbing system. Therefore, in this work, BBSs acted as template/binder for the production of monoliths and as functionalizing agent of the produced monoliths. The reference powders, deeply studied in a published article (Sadraei et al., 2019b), and the monoliths of GAB before and after functionalization were characterized by means of x-ray diffraction to evidence their crystal structure, Fourier transform infrared spectroscopy for evaluating the presence of BBSs on the supports, thermogravimetric analysis to measure the thermal stability of the materials and quantify the functionalizing BBS amount immobilized on the supports, nitrogen adsorption at 77 K for the investigation of the surface area and porosity of the systems, and zeta potential measurements to analyze the effect of BBS immobilization on the surface charge of the supports and to predict the type of interaction, which can be established with substrates. Finally, the systems were applied in removal of pollutants with different charge, polarity, and molecular structure, such as dyes (crystal violet and acid orange 7) and contaminants of emerging concern (carbamazepine and atenolol). Only the cationic dye CV is captured by the adsorbing material, and this allows envisaging a possible use of the functionalized monoliths for selective adsorption of cationic substrates.

## Introduction

Refuse accumulation is a heavy burden for our society, as the normal human activities imply an increasing production of wastes accumulating in dedicated places organized by the municipalities or often abandoned in non-regulated areas. Wastes are generated by domestic, industrial, agricultural, commercial, municipal, and sanitary processes. They can contaminate air, water, and land affecting in turn the health of the biosphere. Part of the problem is faced by a process called waste management, taking into account the management of the already existing refuses, but another complementary approach to reduce the impact of refuses, and the consequent earth pollution, is the Zero Waste Philosophy (https://www.epa.gov/transforming-waste-tool/how-communities-have-defined-zero-waste). It considers that a sustainable life passes through the reduction of wastes and their transformation into raw materials; therefore, it promotes the ideas of recycling and upcycling the waste we produce and increasing the sustainability of the production processes of objects that will be transformed into refuses at the end of their life.

In this perspective, compost is a renewable source of carbonaceous compounds, alternative to the less sustainable petroleum, and bio-based substances (BBSs), isolated from composted urban refuses, are interesting amphiphilic reactants (Montoneri et al., 2008, 2009, 2011). Bio-based substances have been used as binder/templating agents to produce siliceous mesoporous monoliths useful as support for a variety of applications. In 2012, they were employed to support an enzyme in order to obtain a heterogeneous biocatalysts for hydrogen peroxide activation (Magnacca et al., 2012), in this article, the same procedure has been applied to produce alumina-based mesoporous monoliths. In this case, the application of the material was chosen in the field of wastewater remediation, as water contamination, similarly to refuse accumulation, has become a serious worldwide concern that can cause many health problems, particularly in industrial countries. Scientists are facing the issue developing simple, fast, and environmentally friendly methods for decomposition or removal, in general, of pollutants, in particular of organic ones (de Paul Obade and Moore, 2018; Jaramillo and O'Shea, 2018; Vega et al., 2018; Ren et al., 2019).

Among the possible techniques for wastewater treatment, the adsorption process by solid adsorbents demonstrates a high potential as one of the most efficient methods for capturing organic contaminants from wastewaters avoiding the risk of secondary pollution brought by decomposition methods. Several adsorbents, such as activated carbon (Julcour-Lebigue et al., 2012), silica gel (Fan et al., 2012), organic clay (Unuabonah et al., 2013), alumina (Serbezov et al., 2011; Tang et al., 2018; Sadraei, 2019), iron powders (Yu et al., 2013; Zeng et al., 2018), and mesoporous silica (Ko et al., 2014; Ye et al., 2017), have been successfully applied for the removal of dyes from water, but the development of handleable materials that can operate much more easily, in particular in terms of recovery and reusability, is necessary. As the open-framework nature and large pore size (2–50 nm) are the key factors for a good diffusion of the molecules inside the adsorbing materials and a consequent fast adsorption process (Alauzun et al., 2011; Masika and Mokaya, 2011), the production of massive materials possessing these features is pursued, and alumina-based monoliths perfectly fit the requirements.

In addition to this aspect, the adsorption capacity of materials can be enhanced introducing functional groups with high affinity for different substrates by means of functionalization processes. The choice of functional groups allows defining a specific activity of the adsorbing material toward a specific substrate. Oxides carrying OH groups at the surface are very good candidates for being surface-modified, as they can be easily functionalized exploiting several strategies reported in the literature, some of them basing on physical methods, whereas others basing on chemical ones (Nayak et al., 2019; Sadraei et al., 2019a,b; Shanaghi et al., 2019). As in a previous article, we reported the performances of BBS-functionalized alumina powders (Sadraei et al., 2019b). In this work, we are considering the upscaling of the previous study producing mesoporous gamma alumina-based monoliths (GAB-M) functionalized with BBSs (GAB-M–BBSs).

Summarizing, the current research comprehends the following points: (1) the fabrication of new alumina monoliths using BBSs as binder/templating agents, (2) the simple monolith functionalization with BBSs acting as active phase to enhance the adsorption properties, (3) the physicochemical characterization of the produced materials compared with the parent alumina powder, and (4) the adsorption study.

## Materials and Methods

### Materials for Synthesis

Commercial boehmite was kindly supplied by Centro Ricerche FIAT and used to prepare gamma-Al_2_O_3_ (GAB) after calcination in a furnace at 500°C for 3 h, as reported in the literature (Sadraei et al., 2019b).

**Scheme 1:**
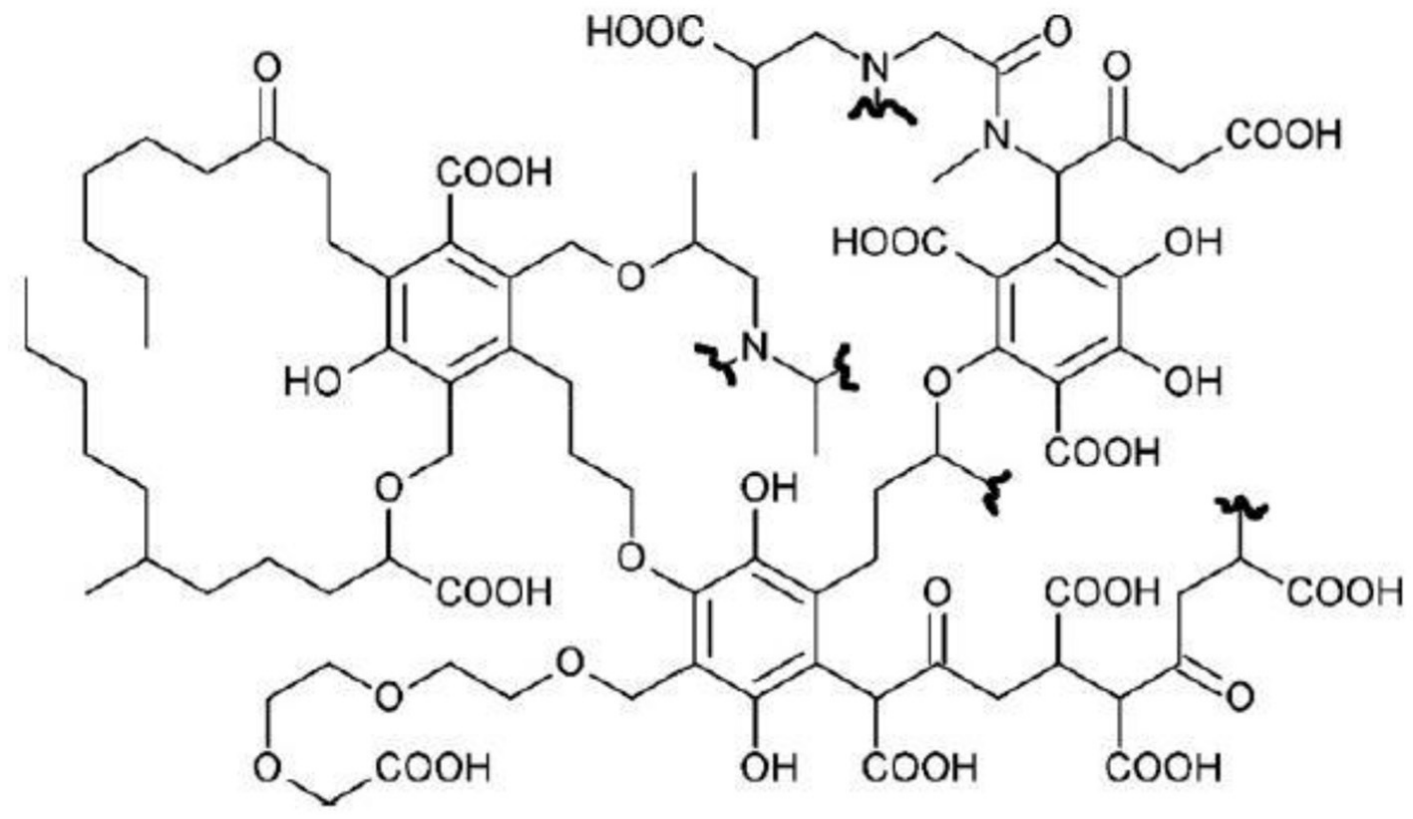
Virtual molecular fragments of BBS.

Bio-based substances were extracted from composted organic refuses aged for more than 180 days supplied by ACEA Pinerolese Industriale. The extraction procedure was described by Montoneri et al. (2008, 2009, 2011). A hypothetic structure of BBS is reported in Scheme 1.

### Materials and Methods for Characterization Study

Scanning electron microscope (SEM) image of the monolith surface was obtained by means of a scanning microscope ZEISS EVO50 XVP equipped with LaB_6_ source and a secondary electron detector. The samples prior to the SEM investigation were sputtered with ~20 nm of a gold layer in order to avoid charging effects using a Bal-tec SCD050 sputter coater.

X-ray diffraction (XRD) analysis was used to investigate the morphology and crystal structure of powders and monoliths. The measurements were performed by using a X'Pert PRO MPD diffractometer from PANalytical, equipped with Cu anode worked at 45 kV and 40 mA in a Bragg–Brentano geometry. In this work, the flat sample-holder configuration was employed.

Fourier transform infrared (FTIR) spectroscopy was applied in a transmission mode by using Bruker Vector 22 spectrophotometer equipped with Globar source and DTGS detector, and working in the transmission mode in the range of 4000–400 cm^−1^ at 4 cm^−1^ resolution. Before investigation all the samples were mixed with KBr powder (1:20 ratio) and pressed to form pellets.

Nitrogen adsorption–desorption measurements at 77 K were carried out by means of ASAP 2020 Micromeritics gas-volumetric apparatus. Prior to the experiments, the samples before and after functionalization were activated at 300 and 40°C, respectively, for 24 h. Specific surface areas were calculated by applying the Brunauer–Emmett–Teller (BET) method (Thommes et al., 2015). Pore volumes and pore size distribution were determined by using the Barrett–Joyner–Halenda method (Thommes et al., 2015) applied to the isotherm adsorption branch.

Thermogravimetric analysis (TGA) was performed by means of TA Q600 (TA Instruments). Materials before and after functionalization were heated at a rate of 10°C/min from 40 to 650°C under air.

Zeta potential (ZP) measurements have been used in order to determine the surface charge of the particles in water at different pH and the point of zero charge of the dispersions (Sadraei et al., 2019b). They were performed using the instrument Zetasizer by Malvern (model ZS90). The suspensions were prepared by mixing 10 mg of sample (after finely crushing in an agate mortar in the case of monoliths) in 20 mL of deionized water under constant stirring (400 rpm) for 15 min. The pH of the suspensions was adjusted in different pH in the range of 3 to 11 by addition of 0.1 M HCl and 0.1 M NaOH solutions. The suspensions were shaken at 25°C temperature for 15 min until the pH had stabilized. A digital pH meter (Metrohm, model 827 pH lab, Swiss mode) was used to measure the pH. In all batch experiments, the refractive index value of alumina was selected.

### Materials and Methods for Adsorption Study

UV-Vis spectrophotometer (Varian Cary 300 Scans) was applied to study the adsorption of the dyes crystal violet (CV, positively charged, maximum absorbance at 584 nm) and acid orange 7 (AO, negatively charged, maximum absorbance at 480 nm) and of the contaminants atenolol (polar, maximum absorbance at 224 nm) and carbamazepine (apolar, maximum absorbance at 284 nm).

The kinetics of the adsorption was carried out contacting 20 mg of adsorbents materials with 10 ppm of contaminant solutions in the total volume of 10 mL at pH 6.5 and keeping under shaking at the temperature of 22 ± 2°C. The removal was evaluated considering the residual contaminant concentrations after separation of the supernatant and measurement of contaminant absorption using a calibration curve.

## Results and Discussion

### Preparation of Materials

#### Preparation of Monoliths as Support

**Figure 1:**
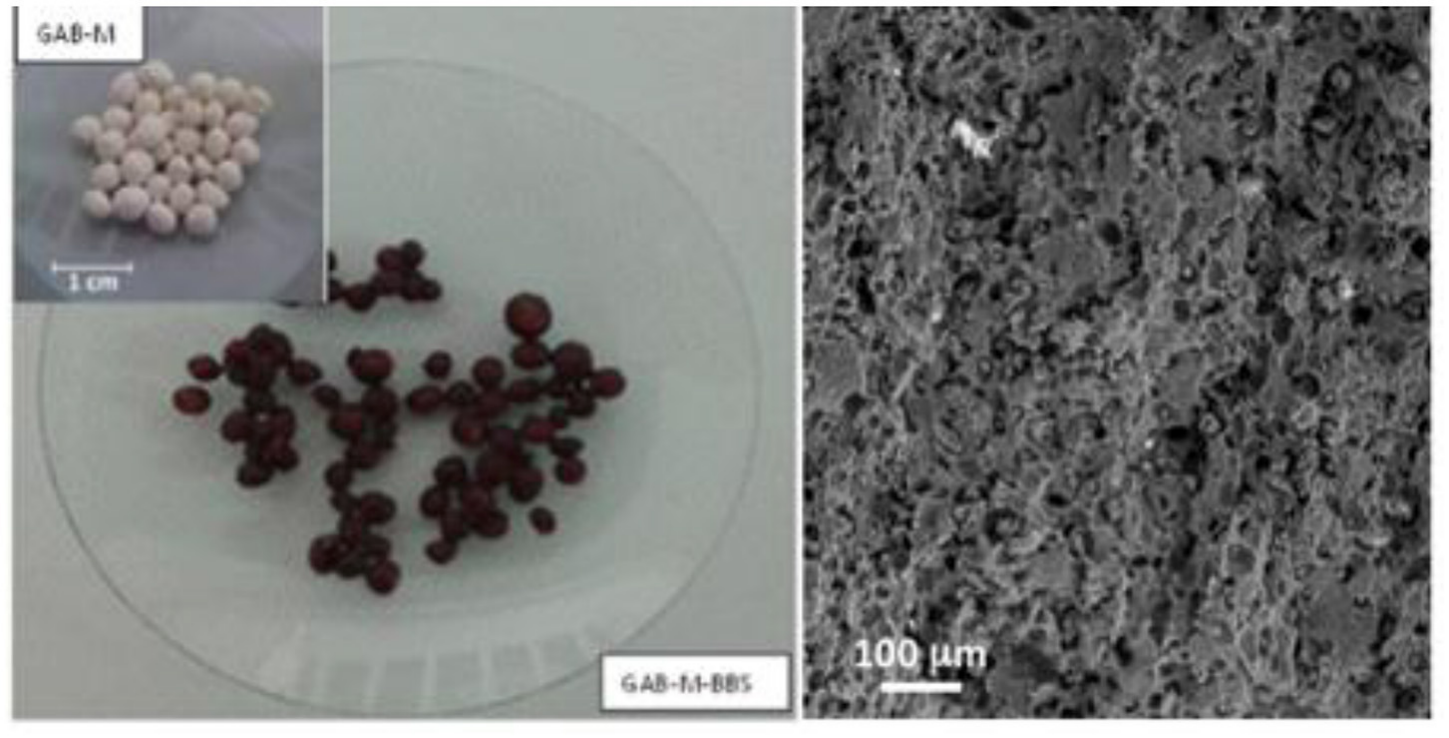
GAB-M before (in the inset) and after functionalization with BBSs (left section) and an SEM image of the monolith surface (right section).

Following the procedure previously applied for preparation of silica monolith (Magnacca et al., 2012), 0.5 g of BBSs was stored under stirring in 7.5 mL water at room temperature (RT) for 2 h. GAB (2 g) was added to the BBS solution. Five milliliters of water was then added, and the mixture was stirred for 2 h. The mixture was left at ambient condition for relaxing and dried at ambient temperature for the time needed to obtain a mud in order to model small spheres of ~0.5 cm of diameter. These spheres were dried overnight at RT and then calcined in furnace at 500°C for 4 h in order to remove all organics and yield a mechanically stable, porous monolith named GAB-M. The image of the spheres after calcination is presented in the inset of Figure 1 together with the SEM image of the monolith surface.

#### BBS Functionalization of Monoliths

As alumina powders were successfully BBS-functionalized, thanks to a simple electrostatic interaction occurring in water at circumneutral pH (Sadraei et al., 2019b), we tried the same procedure to functionalize the surface of GAB-M. The functionalized monoliths were prepared using 1 g of GAB-M dispersed in water at natural pH (~6.5) containing 20 g/L of water-soluble BBSs and two different procedures:

The mixture container was sealed and gently shaken (in order to avoid the monolith breakage) using an orbital mixing plate with rotation at 1000 rpm for 24 h at 25 ± 2°C.

The mixture contained was sealed and, after shaking at RT, was placed in an oven at 60°C overnight and then cooled down and again placed in an oven at 80°C overnight.

At the end of both procedures, the samples were washed with 10 mL of water several times until no signal of leached BBS molecules from GAB-M–BBS samples were evidenced in the UV-Vis spectra of the washing medium. The following drying process was carried out in the oven at 40°C for 24 h.

The first procedure did not allow a complete functionalization of the monolith as the BBS brown color was present only in the outermost layer of the spheres, whereas the second treatment allowed a complete functionalization. The image of the functionalized porous monoliths, named GAB-M–BBSs, is reported in Figure 1. These samples were used for the subsequent characterization, and the powders obtained in the previous work (Sadraei et al., 2019b), GAB and GAB-BBSs, were used as reference materials for comparison.

The preparation route of the non-functionalized monoliths and the following functionalization methods were carried out several times in order to define the reproducibility of the procedures. In all the attempts, the samples presented very similar behaviors, as witnessed by FTIR spectra, XRD, TGA, and N_2_ adsorption/desorption analyses.

### Material Characterization

#### XRD Measurements

**Figure 2:**
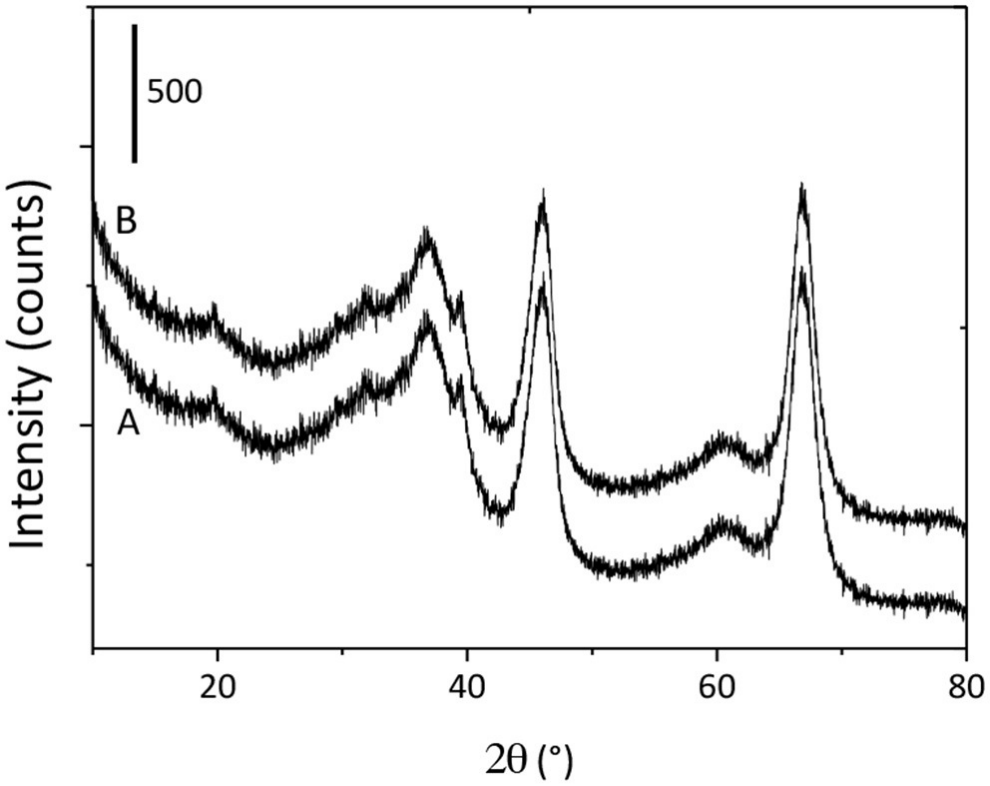
XRD patterns of GAB **(A)** and GAB-M **(B)**.

X-ray diffraction analysis was performed to investigate the effect of monolith fabrication process on the crystal structure of GAB powder. Figure 2 shows the diffraction patterns for both GAB and GAB-M samples. As it can be seen, the crystallographic patterns of the monoliths GAB-M are not different from GAB one. The typical reflections of cubic γ-Al_2_O_3_ [reference pattern 01-075-0921 and (Sifontes et al., 2014)] can be evidenced in both diffractograms; in detail: the signals at 2θ = 39, 46, and 67° corresponding to the reflection of the planes (311), (400), and (440). An analogous curve, not reported for the sake of brevity, can be observed for monoliths after BBS functionalization.

#### FTIR Spectroscopy

**Figure 3:**
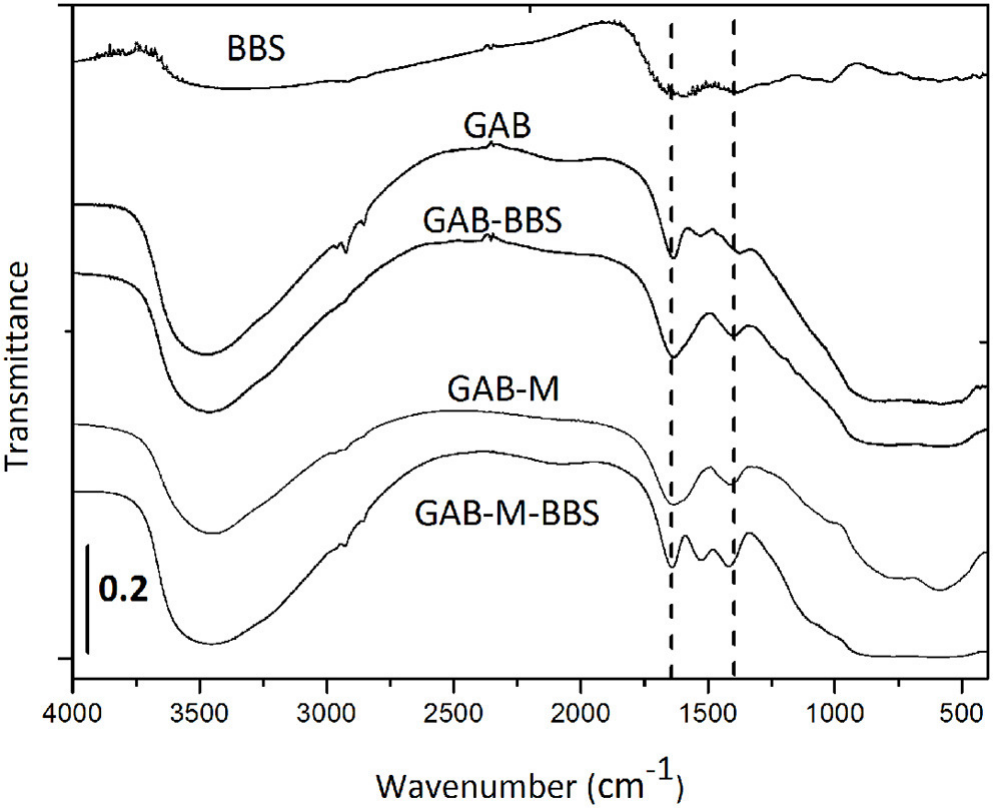
FTIR spectra of sample powders and monoliths before and after functionalization with BBSs. Bio-based substance IR spectrum was reported for the sake of comparison. The curves were shifted for the sake of clarity.

The IR spectra of GAB powder before and after functionalization are reported in Figure 3. The presence of BBSs is clearly visible in the GAB-BBS spectrum. In fact, as for pure BBS sample, it shows the presence of OH groups and atmospheric moisture interacting with the surface and producing a signal at ~3500 cm^−1^ (ν_OH_ vibrations), a large signal at 1600 cm^−1^ due to both carbonylic stretching (ν_C = O_) and vibration of water molecules adsorbed at the surface (δ_HOH_ signal), and other two signals at 1400 and 1000 cm^−1^ due to carboxylic acid/C–H bending and OCO vibrations, respectively. Other weak signals due to ν_CH_ stretching vibrations are visible at around 3000 cm^−1^ (Nisticò et al., 2015; Bianco Prevot et al., 2019; Sadraei et al., 2019b). In addition to these bands, the alumina samples are characterized by a very intense absorption >1000 cm^−1^ due to the bulk vibrations of the solid framework.

The infrared spectra of GAB-M before and after functionalization were collected in the same figure. The spectrum of GAB-M before functionalization is dominated by a very large signal at ~3400 cm^−1^ and by a couple of bands at ~1650 and 1300 cm^−1^. All of them derive from the interaction of the solid samples with the molecules present in the atmosphere, H_2_O and CO_2_. In fact, the burning off of BBSs, in the process of monolith formation, leaves in the material some inorganic residues naturally present in the BBSs (namely, cationic and anionic species such as Mg^2+^, K^+^, Ca^2+^, NO^3−^, Cl^−^), and these species interact very easily with moisture and CO_2_ leading to the formation of intense ν_OH_ signals at high frequency and to the formation of symmetric and antisymmetric vibrations of surface carbonate-like groups at low frequency, respectively. In addition to these absorptions, the BBS functionalization causes the formation of BBS typical bands, as mentioned for GAB-BBS sample.

#### Gas-Volumetric N_2_ Adsorption at 77 K

**Figure 4:**
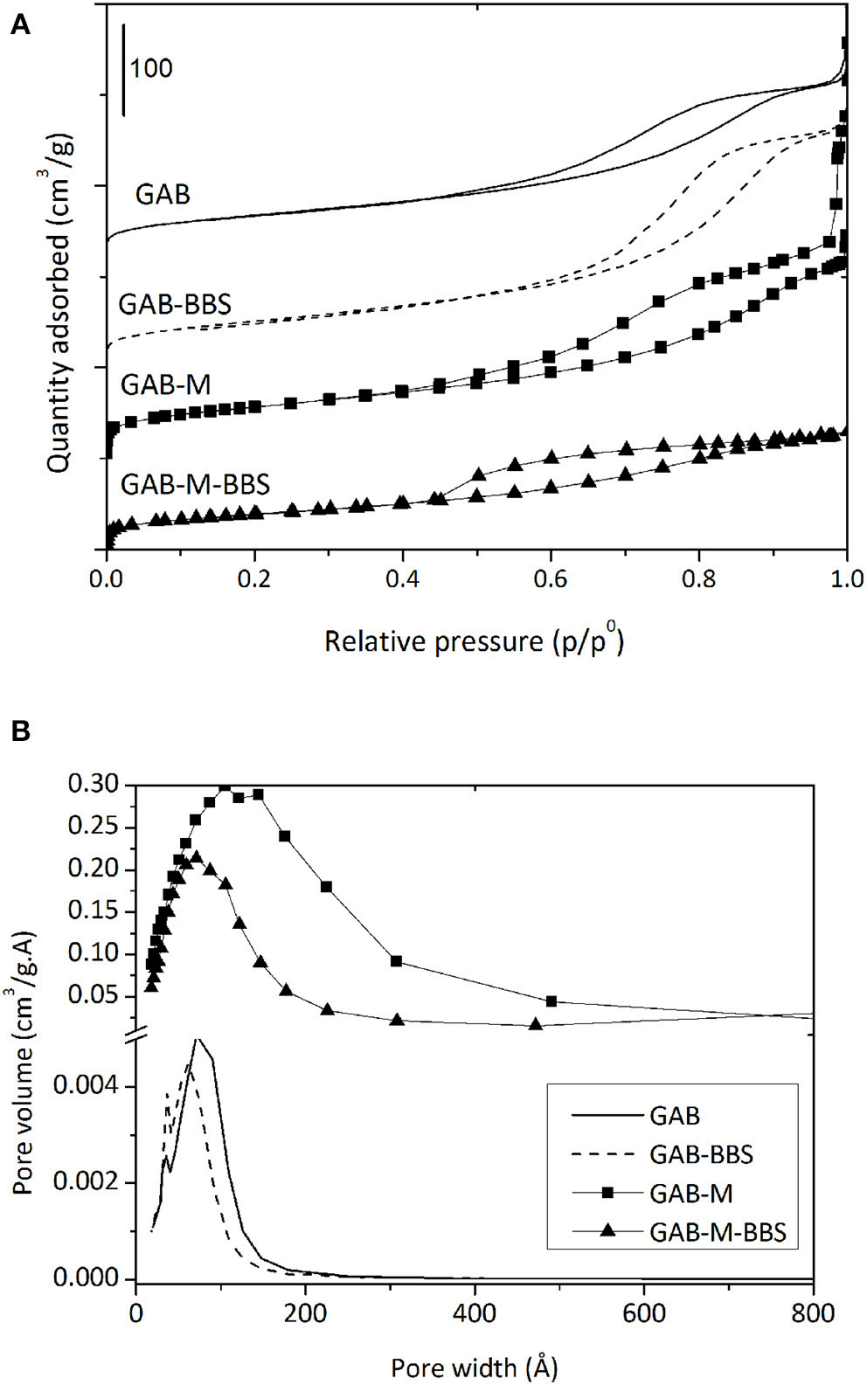
Nitrogen adsorption/desorption isotherms **(A)** and BJH adsorption pore size distribution **(B)** of GAB (solid line), GAB-BBSs (broken line), GAB-M (squares), and GAB-M–BBSs (triangles). In the figure, the curves were shifted for the sake of clarity.

Gas-volumetric analysis of N_2_ adsorbed at 77 K was carried out for all samples including reference powders. According to the International Union of Pure and Applied Chemistry classification, the adsorption/desorption isotherms of all samples, reported in Figure 4A, are of the IV type, with hysteresis loops at relative pressures higher than 0.4, confirming that all materials are mesoporous or even macroporous. The formation of monolith from alumina powder does not affect significantly the specific surface area (198 vs. 186 m^2^/g), but changes significantly the porosity, which appears higher and made up of larger mesopores and macropores of width up to 600 Å with respect to the pure powder (Table 1 and Figure 4B). This effect was already evidenced in the previous work dealing with silica monolith formation from powder (Magnacca et al., 2012), and it is due to the templating effect brought by BBS molecules during alumina particle aggregation.

**Table 1:** Textural features of the samples.

**Materials**	**BET-specific surface area (m^**2**^/g)**	**BJH pore volume (cm^**3**^/g)**
GAB	186	0.42
GAB-BBSs	165	0.29
GAB-M	198	0.32
GAB-M–BBSs	141	0.19

In the case of monolith and reference powder, the functionalization process affects the specific surface area and the mesoporosity of all systems, as BBSs occupy part of the pores (the largest ones) leading to a decrease of the total mesoporosity and consequently to the material surface area. This result confirms the BBS functionalization reaches also the core of the monolith pores, as also suggested by the visual examination of the internal part of the monoliths after functionalization.

#### Thermogravimetric Analysis

**Figure 5:**
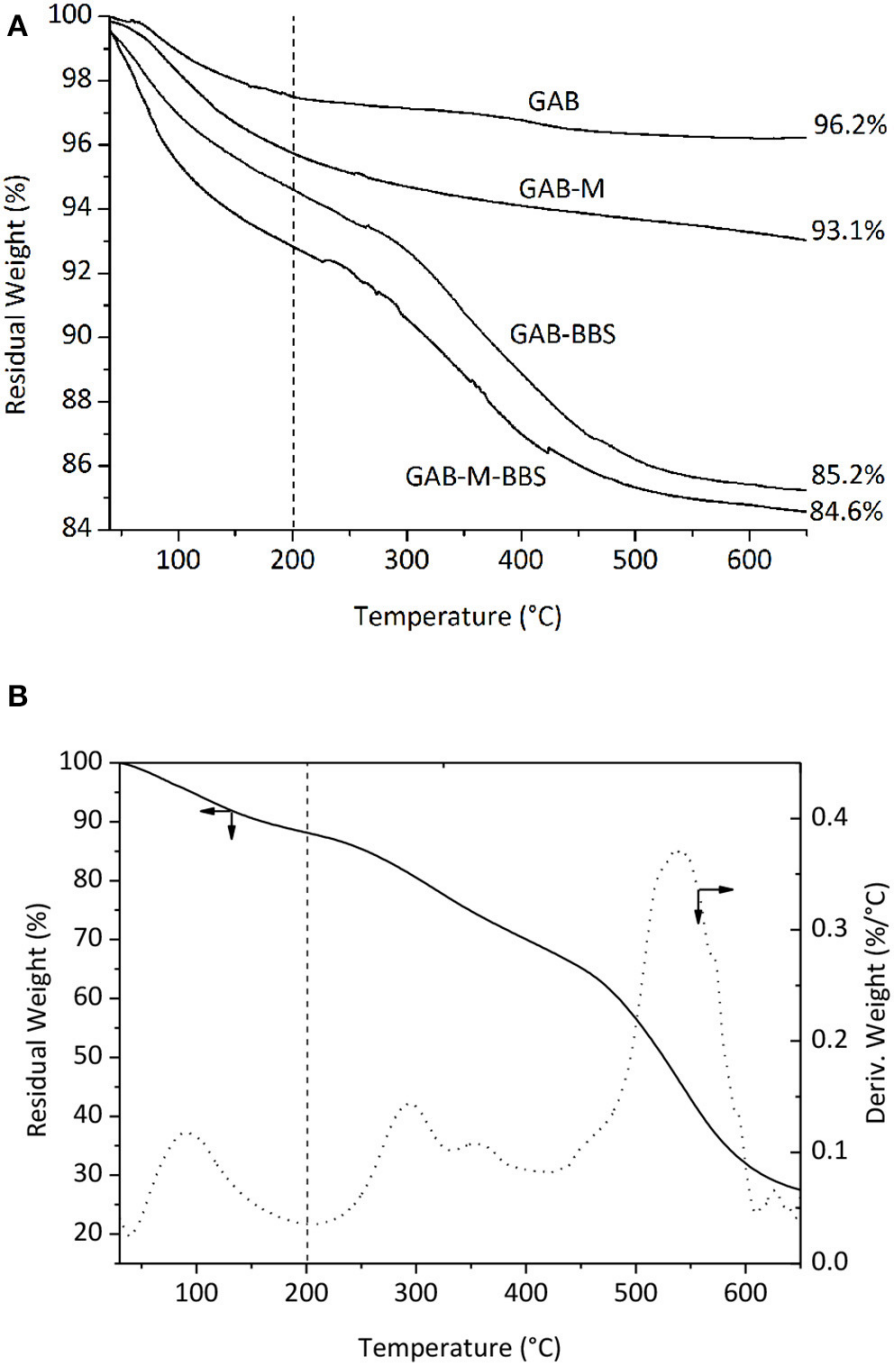
Thermogravimetric analysis curves obtained in air for GAB, GAB-BBSs, GAB-M, and GAB-M–BBSs in **(A)**; weight (solid line) and derivate weight (dotted line) curves of BBSs in **(B)**. The values indicated in the figure represent the residual weight measured for the materials at 650°C; the vertical broken lines indicate the temperature of 200°C used for the quantification of water and organics loss.

The results of TGA analysis on GAB and GAB-M before and after immobilization of BBSs are shown in Figure 5A. Figure 5B shows the curve due to BBS weight loss measured in the same conditions and reports two main regions of weight loss for pure BBS materials, the first one in the range of 40–200°C due to water molecules elimination, and the second one is in the range of 200–650°C due to the oxidation of BBSs. In order to interpret the TGA curve of GAB-BBSs and GAB-M–BBSs, we do need the BBSs, GAB, and GAB-M curves as references. As it can be seen in Figure 5A, all the curves report the two important weight losses described in the case of BBS sample, but while the foster occurs in the range of 40–200°C and is related to the removal of physisorbed and chemisorbed water molecules, the latter one, falling in the range of 200–650°C, is due to a couple of contributions, namely, (i) loss of water obtained from OH groups condensation and (ii) loss of organic matter by oxidation with formation of CO_2_ and H_2_O (Sadraei et al., 2019b). To calculate the amount of organic matter present in the functionalized samples, it is needed to eliminate the contribution due to the condensation of OH groups. The results of this comparison are reported in Table 2. The amount of BBSs loaded onto GAB-BBSs is higher than that observed in the case of GAB-M–BBSs, indicating that a small amount of BBS molecules can be hosted in the mesoporous structure of the monoliths with respect to the parent powder.

**Table 2:** Weight losses observed for references and hybrid materials.

**Materials**	**40–200^**°**^C adsorbed water %**	**200–650^**°**^C organic content and OH groups %**	**OH groups %**	**Measured organic content % ± 0.1**
GAB	2.5	1.3	1.3	–
GAB-BBSs	5.4	9.4	1.3	8.1
GAB-M	4.3	2.6	2.6	–
GAB-M–BBSs	7.2	8.2	2.6	5.6

The amount of water physisorbed and chemisorbed present on the samples and determined by the weight loss in the range 40–200°C confirms that the presence of inorganic residues present on the plain monoliths GAB-M and the additional presence of BBSs present in the functionalized monolith GAB-M–BBSs make the materials much more hydrophilic than the relative powdery ones.

#### Zeta Potential

**Figure 6:**
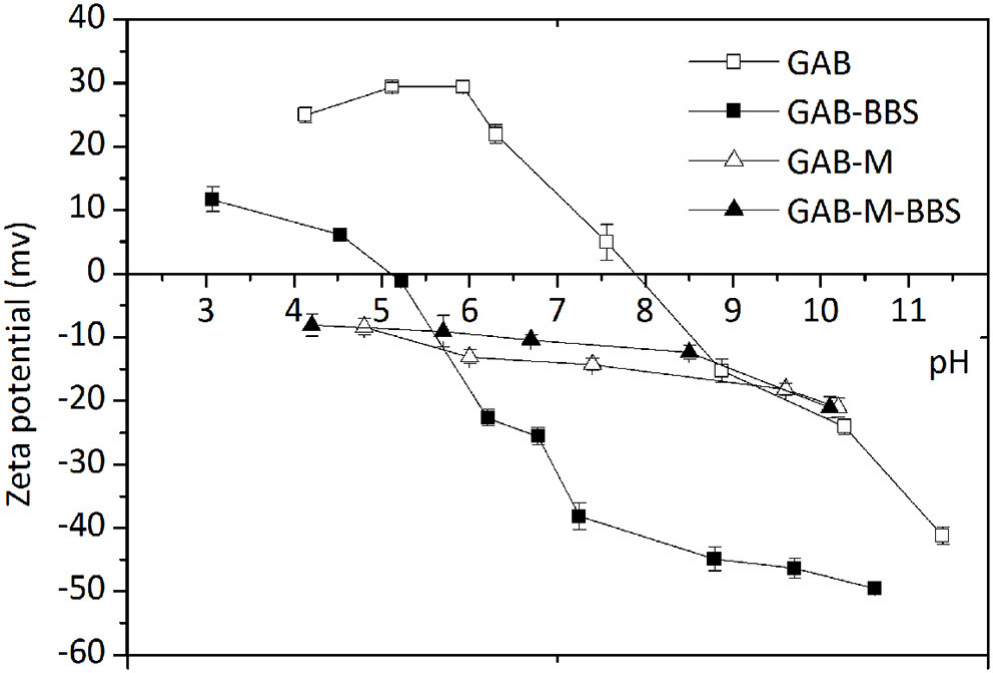
Zeta potential values of GAB, GAB-BBSs, GAB-M, and GAB-M–BBSs as a function of pH. The measurements were carried out on 10 mg of crushed sample dispersed in 20 mL of deionized water. pH was adjusted by addition of HCl or NaOH 0.1 M and stabilized for 15 min.

Zeta potential (ZP) measurements were obtained in order to evaluate the surface charge of the materials before and after immobilization of BBSs, as this is a very useful indication dealing with adsorption process in order to predict the type of substrate suitable for an efficient interaction with the adsorbent. The variations of the ZP values of the samples in the range of pH 3.0–11.0 are reported in Figure 6. GAB possesses a positive surface charge in the range of pH 4–7.9, and then it becomes negative. Water-soluble BBS molecules bring a negative charge at circumneutral pH caused by the presence of dissociated COOH and OH groups (Montoneri et al., 2009); therefore, they can interact quite easily with GAB support. After functionalization, the point of zero charge of GAB-BBS shifts from 7.9 to 5.2, leading to a hybrid material with negatively charged surface at circumneutral pH, prone to the interaction with positive or even partially positive (i.e., polar) substrates (Sadraei et al., 2019b). On the contrary, GAB-M and GAB-M–BBSs show a negative charge in the entire pH range examined, with no substantial modifications brought by BBS functionalization. This feature suggests that only positively charged substrates should interact with these materials.

### Application of Materials in Contaminant Removal

#### Removal of Dyes

**Scheme 2:**
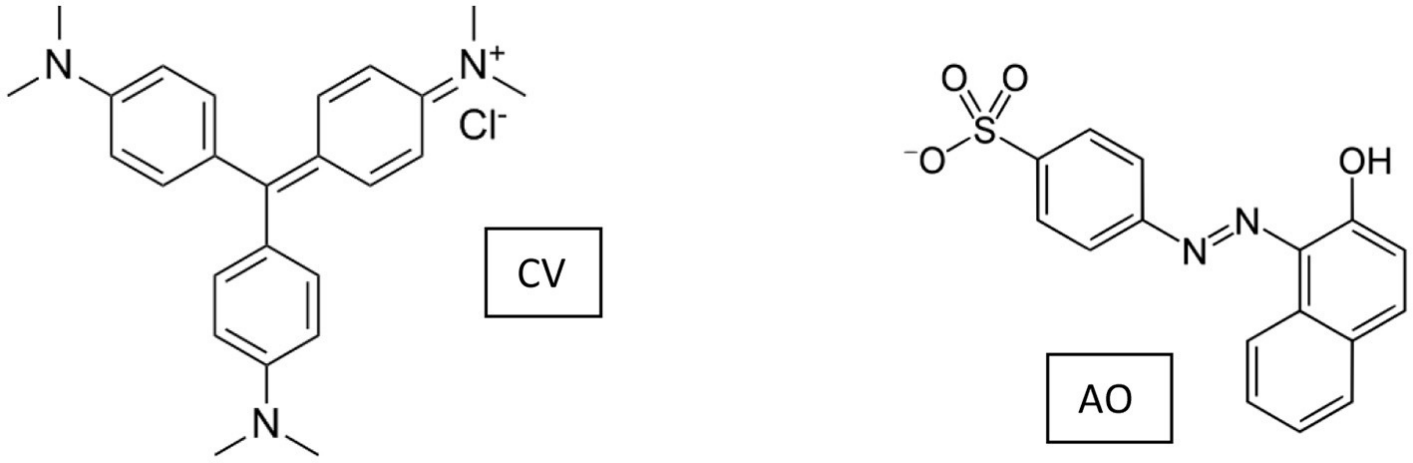
Molecular structure of crystal violet (CV) and acid orange 7 (AO).

Positively charged CV and negatively charged AO (whose molecular structures are shown in Scheme 2) were selected as model adsorptives to evaluate the mechanism at the base of the interaction substrate monoliths. For the sake of comparison, CV adsorption on the reference powdery materials GAB and GAB-BBSs was taken into consideration.

**Figure 7:**
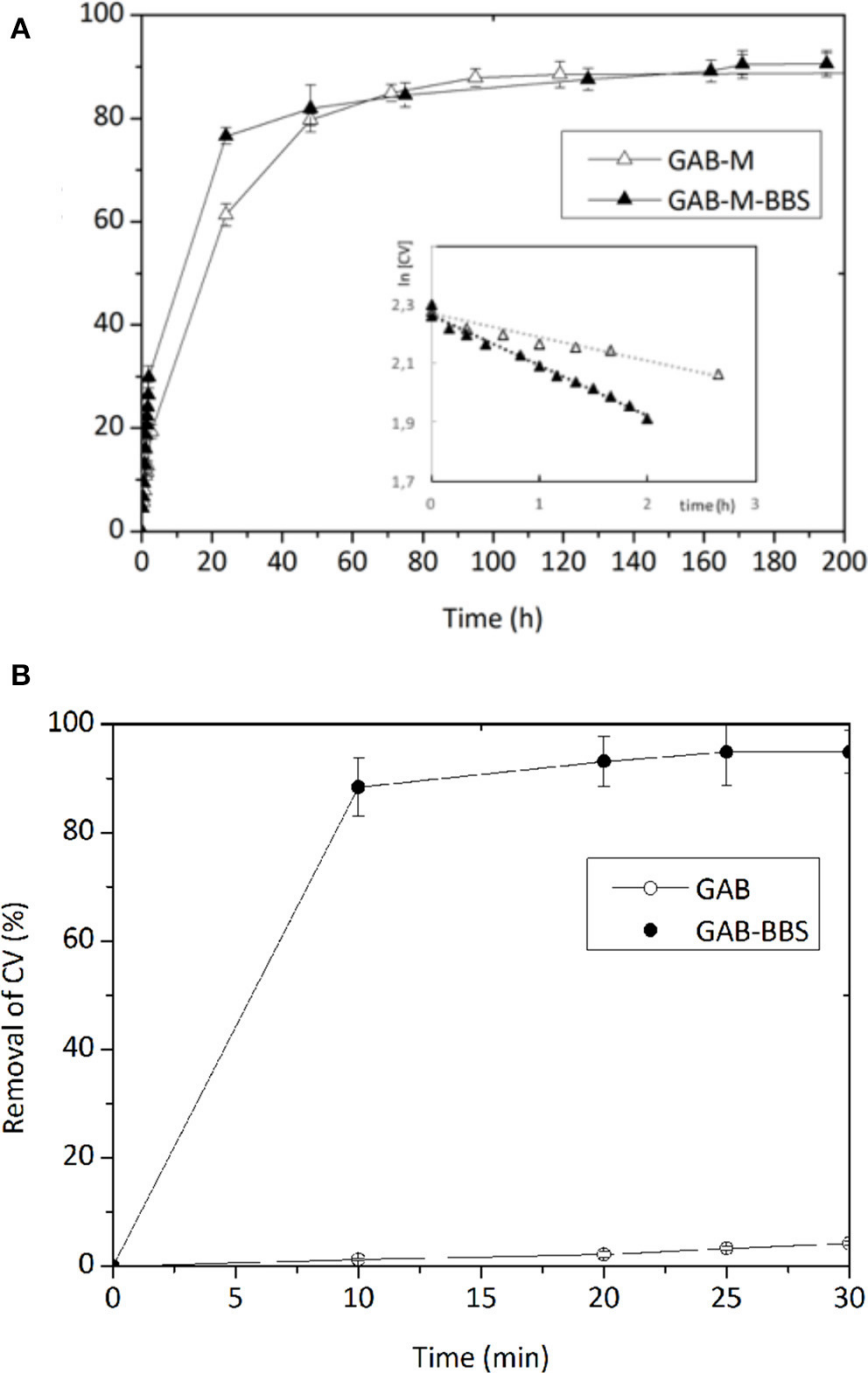
Removal of CV by GAB-M and GAB-M–BBSs **(A)** and by GAB and GAB-BBSs **(B)**. The inset in Section A is the linearized first-order kinetic plot. The measurements were carried out with 20 mg of monolith in 10 mL of CV solution (concentration of 10 mg/L). pH 6.5, *T* = 22 ± 2°C.

Figure 7 shows the adsorption kinetics of CV on the monoliths GAB-M and GAB-M–BBSs (Section A) and on the reference powdery GAB and GAB-BBSs (Section B). No adsorption was evidenced contacting the negatively charged AO with the monolith systems.

As reported in Sadraei et al. (2019b), the adsorption of CV on the powdery GAB-BBSs occurs very quickly and to a large extent, favored by the opposite charges carried by material and substrate. As already evidenced in Sadraei et al. (2019b) and as expected considering the positive charge carried by both material and substrate, the plain GAB powder does not give a good interaction.

The adsorption kinetic curves of CV on GAB-M and GAB-M–BBSs are reported in Figure 7B. In these cases, the interaction occurs in longer time (the experiments took up to 8 days), as the large but flexible CV molecules need to diffuse into the mesopores of monoliths to be adsorbed. Indeed, no significant differences are evidenced for the plain and the functionalized systems in terms of maximum removal, as expected considering the ZP curves and BET measurements discussed in the previous paragraph: both systems are slightly negative and possess similar surface areas; therefore, they can interact almost the same way with the positive substrate. The only change observable is related to the adsorption rate, higher in the case of GAB-M–BBS system, although smaller pores are present in this material with respect to the non-functionalized one. This means that diffusion of dye molecules into the porous adsorbing material is not a steady step for the adsorption process; therefore, we have to consider the high chemical affinity between the negatively charged support and the positively charged CV molecules. In the case of BBSs containing monoliths, the negative charge can be identified with carboxylate groups brought by BBSs, whereas for plain monoliths, a dispersed charge, not localized in one specific site, should be taken into account. In an adsorption process of this kind, a second-order kinetic law is expected, as two species should be responsible of the process, namely, the negative adsorbing surface and the positive dye molecules. In the case of GAB-M–BBS material, it is also possible to try evaluating the relative amounts of adsorbing sites and adsorbed molecules considering the number of carboxylate groups, the number of CV adsorbed molecules, and a stoichiometry of the interaction 1:1. The amount of BBS immobilized on monolith support is 5.6% in weight (as measured by TGA), the corresponding amount of carboxylate groups is 1.40 mmol COOH/g BBSs (Magnacca et al., 2014), and the amount of CV immobilized within 3 h is ~3 ppm, corresponding to ~7 · 10^−8^ mol. We can see that the amount of carboxylic groups available for adsorption is in large excess with respect to the amount of adsorbed dye molecules, so the kinetic law should depend only from CV concentration. In this situation, a kinetic constant of the pseudo–first order could be determined from the kinetic trend. For GAB-M–BBSs, the pseudo *k* = 0.172 h^−1^, whereas for GAB-M, *k*, calculated with analogous procedure, corresponds to 0.080 h^−1^, one-half of the GAB-M–BBS ones. In conclusion, the thermodynamic of the adsorption does not change with or without BBSs, but the BBS presence slightly influences the kinetic of the process.

No AO adsorption was revealed by GAB-M and GAB-M–BBSs, neither for prolonged time of contact, as expected given the negative charge of the dye.

For a comparison with literature data, the CV adsorption capacity of the monoliths was calculated (Liu et al., 2015):

$$\begin{array}{l} q_{t}=\frac{\left(C_{0}-C_{t}\right)\;V}{W} \end{array}$$

In the Equation, *C*_0_ and *C*_t_ (mg/L) are the concentration of CV in solution before and after adsorption, respectively, *V* (L) is the volume of the solution, and *W* (g) is the mass of adsorbents.

As reported in Table 3, the efficiency toward CV adsorption of monoliths studied in this article is similar to or higher than that of other literature adsorbing systems. In principle, therefore, both plain and BBS-functionalized monoliths could be applied to the removal of cationic contaminants. The BBS functionalization, therefore, seems not be functional to the adsorption application. Indeed, this system shows a further advantage with respect to other materials. In fact, it is known that BBSs possess photosensitizing properties (Bianco Prevot et al., 2019), being able of producing Reactive oxygen species (ROS) when irradiated with visible light. The produced ROS (typically the highly reactive OH radicals) can attack and degrade organic molecules until mineralization (complete abatement with formation of CO_2_ and H_2_O), with consequent increase of the applicative interest of this material. The challenge, so far, is the possibility of irradiating all the BBS molecules present in the material, including the molecules immobilized inside the porosities of the monolith, in order to achieve a high abatement efficiency. Thus, once a suitable shape of the support is elaborated, development of the material in photocatalytic applications could be pursued.

**Table 3:** Efficiency of various adsorbents toward CV removal from aqueous solutions.

**Adsorbent**	**Amount adsorbed (mg/g)**	**References**	**Experimental condition (pH, initial concentration, temperature)**
Multiwalled carbon nanotubes	4.48	Gabal et al., 2014	pH 8 Concentration: 5 mg/L Temperature: 25–50°C
*Calotropis procera* leaf	4.14	Ali and Shah, 2008	pH 5 Concentration: 25 mg/L Temperature: 20°C
CaCO_3_ bare	3.35	Liu et al., 2015	pH 6 Concentration: 40 mg/L Temperature: 25°C
Unexpanded perlite	3.30	Dogan and Alkan, 2003	pH 11 Concentration: 122 mg/L Temperature: 30 ± 1°C
Coir pith	2.56	Namasivayam et al., 2001	pH 6.5 Concentration: 40 mg/L Temperature: 30 ± 2°C
Expanded perlite	1.14	Dogan and Alkan, 2003	pH 11 Concentration: 122 mg/L Temperature: 30 ± 1°C
*GAB-M/GAB-M–BBSs*	*4.48*	This work	pH 6.5 Concentration: 10 mg/L Temperature: 22 ± 2°C

#### Removal of Contaminants of Emerging Concern

**Scheme 3:**
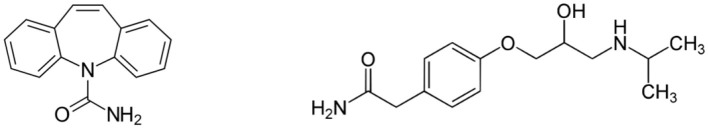
Molecular structure of carbamazepine (left side) and atenolol (right side).

The molecular structure of the contaminants analyzed is reported in Scheme 3.

Contrary to what happened with reference powders and reported in Sadraei et al. (2019b), atenolol (polar non-charged substrate) and carbamazepine (essentially apolar substrate) did not give valuable adsorption on monoliths, even if the large pores of these systems should favor the diffusion and consequent adsorption of the substrates. Probably the simple van der Waals forces that could establish between the poorly charged monoliths and the non-charged substrates are not enough to produce a stable interaction.

## Conclusions

γ-Alumina–based monoliths were prepared following a previously developed procedure (Magnacca et al., 2012). The results showed that they can be prepared in a reproducible way, with good mechanical property, large mesoporosity/macroporosity, and a surface area similar to the parent powdery system. With respect to the pure powder, the monoliths contain some inorganic residues deriving from the BBSs used as template/binder that remain in the alumina framework after the calcination was performed to remove the organic matter and obtain the monoliths. The inorganic residues are responsible of the interaction with atmospheric moisture and CO_2_, the last forming carbonate-like species on the monolith surface, and of the slightly negative surface charge of the material.

Bio-based substance molecules were also used to functionalize the alumina monolith in order to reproduce the results obtained with parent alumina powder and obtain a good adsorbent for polar pollutants, handleable, and therefore useful for upscaling the material to real application. The interaction of the functionalized monoliths with positive, negative, polar non-charged, and apolar molecules (chosen in the family of dyes and contaminants of emerging concern) evidenced a good interaction with positively charged species, opening the way to the use of the BBS-containing monoliths for the selective capture of pollutants based on their charge.

## Data Availability Statement

All datasets generated for this study are included in the article/supplementary material.

## Author Contributions

GM followed the experiments and revised the manuscript. FN carried out the experiments and most part of the data elaboration. RS helped in carrying out the experiments and wrote the manuscript. All authors contributed to the article and approved the submitted version.

## Conflict of Interest

The authors declare that the research was conducted in the absence of any commercial or financial relationships that could be construed as a potential conflict of interest.
